# Fingerling stocking size has no influence on proliferative gill disease severity in farm-raised Channel Catfish

**DOI:** 10.1093/jahafs/vsae002

**Published:** 2025-04-04

**Authors:** Bradley M Richardson, Noor ul-Huda, Cynthia Ware, Alvin C Camus, Caitlin E Older, Fernando Y Yamamoto, Penelope M Goodman, J Grant Reifers, Charles M Walker, Justin M Stilwell, David P Marancik, David J Wise, Matt J Griffin

**Affiliations:** U.S. Department of Agriculture, Agricultural Research Service, Warmwater Aquaculture Research Unit, Stoneville, Mississippi, USA; Department of Pathobiology and Population Medicine, College of Veterinary Medicine, Mississippi State University, Mississippi State, Mississippi, USA; Thad Cochran National Warmwater Aquaculture Center, Delta Research and Extension Center, Mississippi State University, Stoneville, Mississippi, USA; Department of Pathobiology, School of Veterinary Medicine, St. George’s University, True Blue, Grenada, West Indies; Department of Pathobiology and Population Medicine, College of Veterinary Medicine, Mississippi State University, Mississippi State, Mississippi, USA; Thad Cochran National Warmwater Aquaculture Center, Delta Research and Extension Center, Mississippi State University, Stoneville, Mississippi, USA; Department of Pathology, College of Veterinary Medicine, University of Georgia, Athens, Georgia, USA; U.S. Department of Agriculture, Agricultural Research Service, Warmwater Aquaculture Research Unit, Stoneville, Mississippi, USA; Thad Cochran National Warmwater Aquaculture Center, Delta Research and Extension Center, Mississippi State University, Stoneville, Mississippi, USA; Department of Wildlife, Fisheries and Aquaculture, College of Forest Resources, Mississippi State University, Mississippi State, Mississippi, USA; Thad Cochran National Warmwater Aquaculture Center, Delta Research and Extension Center, Mississippi State University, Stoneville, Mississippi, USA; Department of Wildlife, Fisheries and Aquaculture, College of Forest Resources, Mississippi State University, Mississippi State, Mississippi, USA; Thad Cochran National Warmwater Aquaculture Center, Delta Research and Extension Center, Mississippi State University, Stoneville, Mississippi, USA; Department of Wildlife, Fisheries and Aquaculture, College of Forest Resources, Mississippi State University, Mississippi State, Mississippi, USA; Thad Cochran National Warmwater Aquaculture Center, Delta Research and Extension Center, Mississippi State University, Stoneville, Mississippi, USA; Department of Wildlife, Fisheries and Aquaculture, College of Forest Resources, Mississippi State University, Mississippi State, Mississippi, USA; Department of Pathobiology and Population Medicine, College of Veterinary Medicine, Mississippi State University, Mississippi State, Mississippi, USA; Department of Pathobiology, School of Veterinary Medicine, St. George’s University, True Blue, Grenada, West Indies; Thad Cochran National Warmwater Aquaculture Center, Delta Research and Extension Center, Mississippi State University, Stoneville, Mississippi, USA; Department of Wildlife, Fisheries and Aquaculture, College of Forest Resources, Mississippi State University, Mississippi State, Mississippi, USA; Department of Pathobiology and Population Medicine, College of Veterinary Medicine, Mississippi State University, Mississippi State, Mississippi, USA; Thad Cochran National Warmwater Aquaculture Center, Delta Research and Extension Center, Mississippi State University, Stoneville, Mississippi, USA

**Keywords:** aquaculture, disease, *Henneguya ictaluri*, myxozoan, parasite, proliferative gill disease

## Abstract

**Objective:**

The myxozoan *Henneguya ictaluri* is the causative agent of proliferative gill disease (PGD) in Channel Catfish *Ictalurus punctatus* and hybrid catfish (Channel Catfish × Blue Catfish *I. furcatus*), which is a significant disease concern within the commercial catfish industry of the southeastern United States. Incidence of PGD occurs most frequently in fingerling-sized catfish when the fish are being transferred from nursery ponds to grow-out ponds. Mitigation strategies for PGD primarily involve the avoidance of stocking fish into ponds with existent lethal concentrations of the parasite, as determined through sentinel fish exposures or *H. ictaluri-*specific quantitative PCR. This study aimed to evaluate the potential of stocking larger fingerlings to improve survival and investigate the influence on three metrics of gill condition.

**Methods:**

Two sizes of Channel Catfish fingerlings (∼12 and ∼20 cm) were stocked into nylon-mesh net-pens located in 19 commercial ponds with varying levels of *H. ictaluri* activity. After 1 week, fish were removed from the ponds and mortality was recorded. All survivors were euthanized for gross, histological, and molecular assessment. Gill biopsies from surviving fish were evaluated to estimate gill damage based on the presence of chondrolytic lesions in gill clip wet mounts. The number of characteristic PGD lesions and the number of presporogonic stages present were assessed histologically.

**Results:**

Generalized linear regression showed no interaction between parasite burden in the pond water or gill tissues and fingerling size. In all regressions, only parasite concentrations in pond water or gill tissues were significant predictors of any gill condition metrics.

**Conclusions:**

This study suggests that stocking of larger fingerlings provides no appreciable protection from PGD mortality or sublethal gill damage. Though smaller fingerlings regularly showed slightly better average gill condition compared to larger fingerlings, this occurred primarily in ponds with the highest parasite concentrations, which were likely influenced by survival bias.

## INTRODUCTION

*Henneguya ictaluri* (Cnidaria: Myxobolidae) is the causative agent of proliferative gill disease (PGD) in Channel Catfish *Ictalurus punctatus* and hybrid catfish (Channel Catfish × Blue Catfish *I. furcatus*; Bosworth et al., 2003; Griffin et al., 2010; Pote et al., 2000, 2012). Proliferative gill disease is the most common parasitic disease in diagnostic case submissions to the Aquatic Research and Diagnostic Laboratory at the Thad Cochran National Warmwater Aquaculture Center in Stoneville, Mississippi, accounting for approximately 10–20% of all case submissions annually. Initial penetration and proliferation of the actinospore stage in the gills of Channel Catfish and hybrid catfish result in a proliferative and destructive branchial disease characterized by severe granulomatous inflammation, epithelial hyperplasia that fills interlamellar spaces, and lysis of gill filament cartilage (Griffin et al., 2010; Rosser et al., 2019; Wise et al., 2008). Currently available treatments for PGD are strictly palliative and of limited effectiveness in severe outbreaks (Wise et al., 2004). Consequently, industry practices for mitigating morbidity and mortality associated with PGD outbreaks are largely limited to increased aeration (Pote et al., 2012).

The life cycle of *H. ictaluri* is typical of other myxozoans, consisting of a myxospore stage that matures in Channel Catfish and a pelagic actinospore stage released by the benthic oligochaete *Dero digitata*, which is ubiquitous across catfish operations in the southeastern United States (Burtle et al., 1991; Pote et al., 2000; Styer et al., 1991). Prolonged exposure of Channel Catfish and hybrid catfish to the actinospores results clinically in inappetence; morbidity; and, in severe cases, death (Wise et al., 2004, 2008). The disease is most prevalent in the spring, with lesser incidence in the fall, predicated by *D. digitata* population dynamics (Wise et al., 2008). Outbreak severity is positively correlated with parasite levels in pond water, which can be estimated by quantitative PCR (qPCR; Griffin et al., 2009). Management strategies to minimize oligochaete populations in the pond would in turn reduce the number of potential hosts available to release infective actinospores into the water, indirectly reducing the incidence and severity of PGD (Tucker & Hargreaves, 2004). However, chemical therapeutics and biological control measures targeting the oligochaete host in pond muds have been largely unsuccessful (Mischke et al., 2001, 2014, 2016).

At present, industry practices to minimize PGD losses revolve around sentinel fish exposures or, in some cases, molecular assays to assess parasite concentrations in pond water prior to stocking (Griffin et al., 2009; Wise et al., 2004, 2008). Although removing fish from infectious water can allow fish to recover (Wise et al., 2008), this incurs a cost to farmers and can stress fish. Therefore, selectively stocking ponds with low PGD risk to prevent significant losses is a more practical method. Sentinel fish can be used to estimate actinospore levels (Wise et al., 2004, 2008); however, an *H. ictaluri-*specific qPCR, which requires less labor and time, was shown to correlate well with sentinel fish data (Griffin et al., 2009) and has been implemented by several producers to dictate their springtime stocking schedules. Another potential method of controlling PGD losses on commercial operations comes from work indicating that hybrid catfish are a dead-end host for *H. ictaluri* and that stocking choice—Channel Catfish versus hybrid catfish—influences myxozoan community dynamics (Griffin et al., 2020; Stilwell, Camus, Ware et al., 2023; Stilwell, Camus, Woodyard et al., 2023). Although infection occurs in hybrids, the development of presporogonic stages is arrested (Rosser et al., 2019; Stilwell, Camus, Ware et al., 2023; Stilwell, Camus, Woodyard et al., 2023). As a result, utilization of a “crop rotation” system, similar to rotational grazing strategies proposed to minimize roundworm infections in livestock (Rocha et al., 2008), is being evaluated as a means to suppress *H. ictaluri* actinospore concentrations and reduce PGD outbreaks.

The potential use of larger fingerlings for stocking as a means of limiting mortalities was proposed by farmers to the staff at the Thad Cochran National Warmwater Aquaculture Center and was tested in the present study. The hypothesis follows that larger fish might withstand a higher parasite burden in terms of anatomical damage and survival than smaller fish. Intuitively, the greater gill surface area in larger fish suggests that more organisms would be required to cause the same relative amount of damage as a lower parasitic load in the gills of smaller fish. However, more available gill tissue in larger fish might result in greater infection susceptibility by providing a greater surface area for parasite attachment. In contrast, the higher rates of mass-specific oxygen consumption by smaller fish (Andrews & Matsuda, 1975) suggest that less respiratory damage would be required to produce the same level of physiological stress. As both arguments are biologically plausible, it is important to understand the differences in PGD risk between the two sizes of fingerlings as a means of mitigating losses in the catfish industry.

## METHODS

### Study design

In May 2022, two sizes of Channel Catfish fingerlings were stocked into net-pens (10 fish/cage) in each of 19 ponds across 2 weeks; eight ponds were stocked in week 1, and 11 additional ponds were stocked in week 2. In commercial practice, fingerlings are typically sold by weight categories; thus, the fish size-classes used in this study correlate to industry-relevant fingerling weights of 18.14 kg (40 lb) per 1,000 (“small”; 14.7 ± 0.53 cm [mean ± SD]) and 54.43 kg (120 lb) per 1,000 (“large”; 19.4 ± 1.33 cm [mean ± SD]). Fish were maintained in each pond for 7 d, and survivorship was assessed at harvest. Pond water, which was collected on the days of stocking and harvest, was used to assess *H. ictaluri* actinospore concentrations using qPCR analysis (Griffin et al., 2009). Briefly, a 500-mL sample of pond water was collected adjacent to the sentinel cages and approximately 15.24–30.48 cm (6–12 inches) below the surface. Samples were concentrated by centrifugation at 9,500 × *g* for 10 min, and the supernatant was removed by gentle decanting. The pellet was resuspended in 30 mL of distilled water; transferred to a 30-mL, round-bottomed centrifuge tube; and concentrated at 20,000 × *g* for 10 min. The supernatant was removed, the pellet was resuspended in 1 mL of distilled water and transferred to a PowerBead for processing (DNeasy Powersoil Pro Kit; Qiagen, Hilden, Germany), and *H. ictaluri* actinospore equivalents were estimated by qPCR following the procedures of Griffin et al. (2008, 2009).

Surviving catfish were euthanized by immersion in an overdose of tricaine methanesulfonate (MS-222; >300 mg/L), and gills were removed aseptically for further evaluation. Tissues for molecular and gross analysis were excised from the left operculum, while gill arches (*n* = 4 arches/fish) for histopathology were taken from the right operculum. Gill clip biopsies (∼40–80 filaments) designated for molecular analysis were placed in 600 µL of cell lysis solution, DNA was extracted using a Puregene DNA isolation kit (Qiagen) based on protocols by Griffin et al. (2008), and isolated DNA standardized to 10 ng/μL with Buffer EB and 50 ng of total DNA was used as template in the qPCR. Gills underwent gross examination on wet mounts to quantify the percentage of filaments with chondrolysis following the procedures set forth by Wise et al. (2008), evaluating approximately 30–40 filaments from each fish.

Whole gill arches for histological evaluation were placed in 10% neutral buffered formalin and were processed for histopathological evaluation using standard methods, followed by staining with hematoxylin and eosin. Gills were assessed by counting the number of lesions and presporogonic stages present in 10 of the longest sequential gill filaments on three separate arches per fish. Lesions were defined as the presence of inflammation within the gill filament with or without cartilage breaks or presporogonic stages, hemorrhage, cartilage breaks, and/or regenerative chondrodysplasia.

The presence of *H. ictaluri* presporogonic stages was confirmed in select gill sections using an in situ hybridization assay specifically developed for *H. ictaluri* with RNAscope technology (Advanced Cell Diagnostics, Inc., Newark, California; Stilwell, 2021; Stilwell, Camus, Ware et al., 2023; Stilwell, Camus, Woodyard et al., 2023). In brief, unstained histologic sections were prepared on charged slides, dried, treated with hydrogen peroxide, rinsed in distilled water, and immersed in target retrieval solution. Once removed, slides were dried overnight before being subjected to protease and probe hybridization steps, followed by signal amplification and detection steps using an RNAscope HybEZ oven (Advanced Cell Diagnostics). Finally, slides were counterstained with hematoxylin and Tris-buffered saline (TBS; bluing reagent), dried, and cover-slipped using EcoMount (Biocare Medical LLC, Concord, California).

### Analyses

All statistical analyses were performed using program R version 4.3.0 (“Already Tomorrow”; R Core Team, 2023). Survival was evaluated using a two-parameter binomial dose–response model with the drm function within the package drc version 3.0.1 (Ritz et al., 2015). Dose–response models were created using qPCR quantification cycle (Cq) values in the pond water as the predictor variable. The Cq values represent the log-transformed estimates of DNA equivalents within a sample; thus, these values were favored for analysis over actual estimated DNA quantities because they remain on a consistent scale compared to the large variation in magnitude of DNA estimates. The model was used to estimate dose responses for each fish size-class (small vs. large), yielding an estimate of the response slope and the concentration that was lethal to 50% of test organisms (LC50) for each.

Gill damage, number of lesions, and number of presporogonic stages were analyzed using a generalized mixed-effects model with a negative binomial distribution (glmer.nb) in the package lme4 version 1.1.31 (Bates et al., 2015). The Cq value from the pond water or gill tissue qPCR, fish size, and their interaction were used as fixed effects, while pond number was included as a random variable. Results from the models were considered significant based on an α value of 0.05.

## RESULTS

Results from a rate ratio test showed no significant difference (*P* = 0.89) between the mortality incidence rate in ponds stocked during week 1 (0.58; 95% CI = 0.46–0.71) and those stocked during week 2 (0.59; 95% CI = 0.49–0.70). The estimated incidence rate ratio between the 2 weeks was 0.98 (95% CI = 0.74–1.29). Based on these results, the data from both week-long trials were combined for analysis.

### Survival

**Figure 1.**
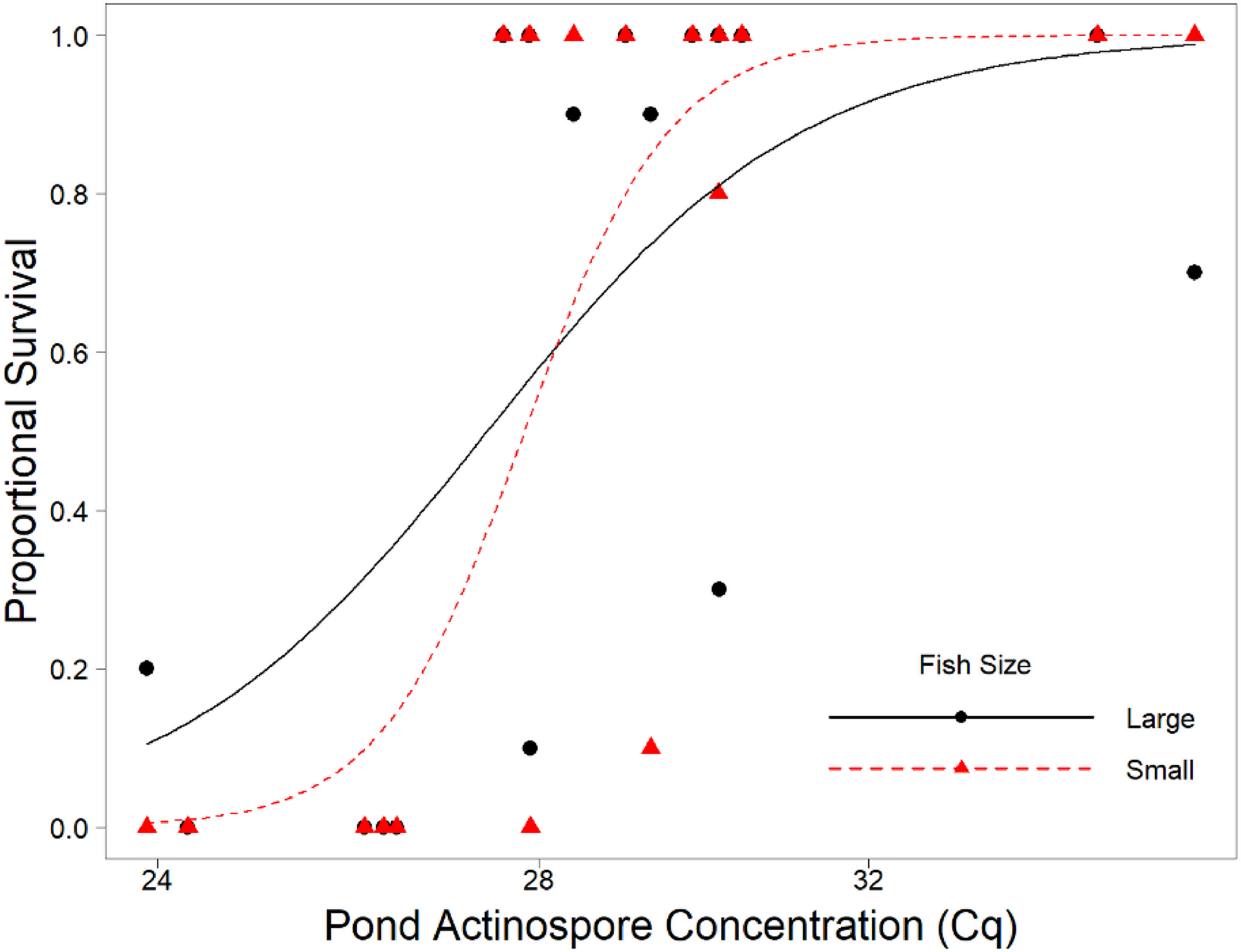
Binomial dose–response curve based on *Henneguya ictaluri* actinospore concentrations in pond water. Response curves represent large (black circles with solid line) and small (red triangles with dashed line) Channel Catfish fingerlings. The quantitative PCR quantification cycle (Cq) values have an inverse relationship with estimated DNA concentration.

The response slope of small and large fingerlings differed significantly (*P* < 0.001). Small fish showed a significantly sharper slope (estimate ± SE = 12.55 ± 2.04) compared to large fish (8.63 ± 1.66); however, the LC50 estimates for the two size-classes were similar, with Cq values of 27.00 ± 0.38 and 26.53 ± 0.53 for small and large fish, respectively (Figure 1).

In addition to the dose–response curve, a generalized linear mixed-effects model with a binomial distribution was also performed to evaluate the influence of pond water parasite load, fish size, and their interaction on the odds of mortality. Results showed a significant interaction between pond water parasite concentration (as Cq value) and fish size (*z* = 2.303, *P* = 0.02). The model provided a survival odds ratio of 1.10 (95% CI = 1.01–1.19) in favor of small fish, suggesting that for a given parasite load in the water, small fish had a slightly higher odds of survival compared to their larger counterparts.

### Effects of parasite burden

**Figure 2.**
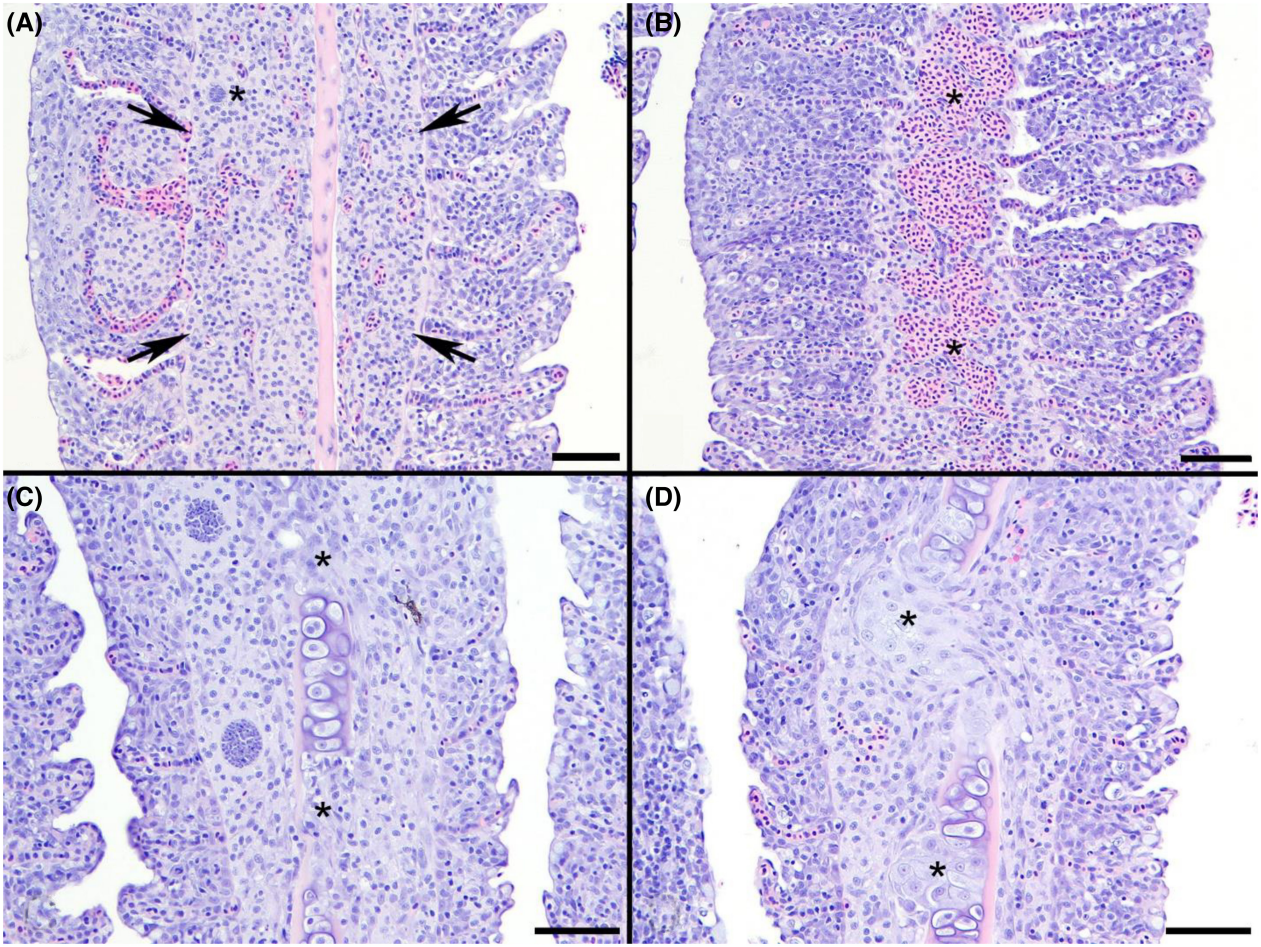
Lesion criteria used in histologic scoring of proliferative gill disease lesions in Channel Catfish: (A) inflammatory cell infiltrates expanding the gill filament with or without cartilage breaks or presporogonic stages (arrows); (B) congestion within gill filaments (asterisks); (C) focal chondrolysis (cartilage breaks) of the gill filaments (asterisks); and (D) abnormal regeneration (chondrodysplasia) of filament cartilage (asterisks). Scale bars represent 50 µm.

**Figure 3.**
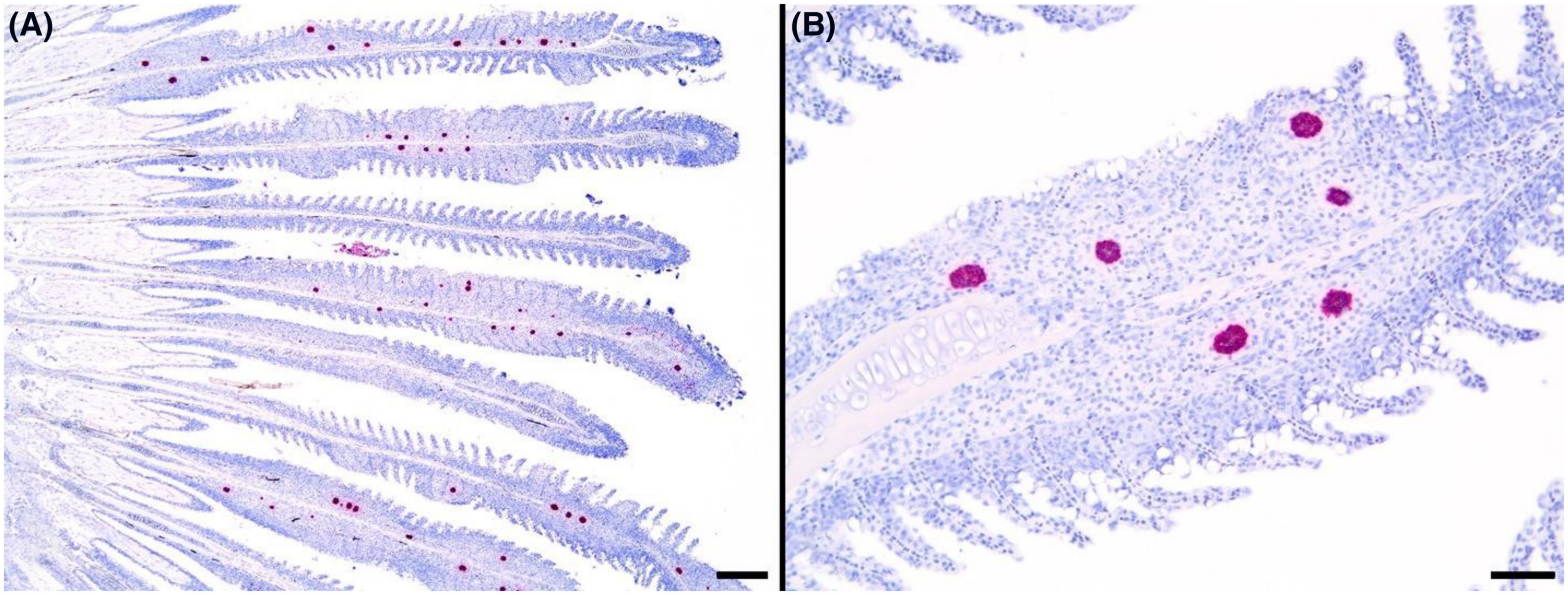
Images from the *Henneguya ictaluri-*specific in situ hybridization assay from select Channel Catfish gills. Presporogonic stages of *H. ictaluri* are represented by red and dark-colored aggregations within the cartilage and filaments of the gill tissues. Significant swelling of the primary and secondary lamellae is also evident in many of the infested tissues. Scale bars for panels A and B are 200 and 50 µm, respectively.

Histologically, PGD is characterized by multifocal combinations of gill filaments expanded by intense granulomatous inflammatory cell infiltrates, epithelial hyperplasia filling interlamellar troughs, congestion, lysis of filament cartilage, and the presence of presporogonic stages within areas of inflammation (Figure 2). Presporogonic stages found in the gill tissues were confirmed as *H. ictaluri* by using the species-specific in situ hybridization assay on the gill tissues (Figure 3).

**Figure 4.**
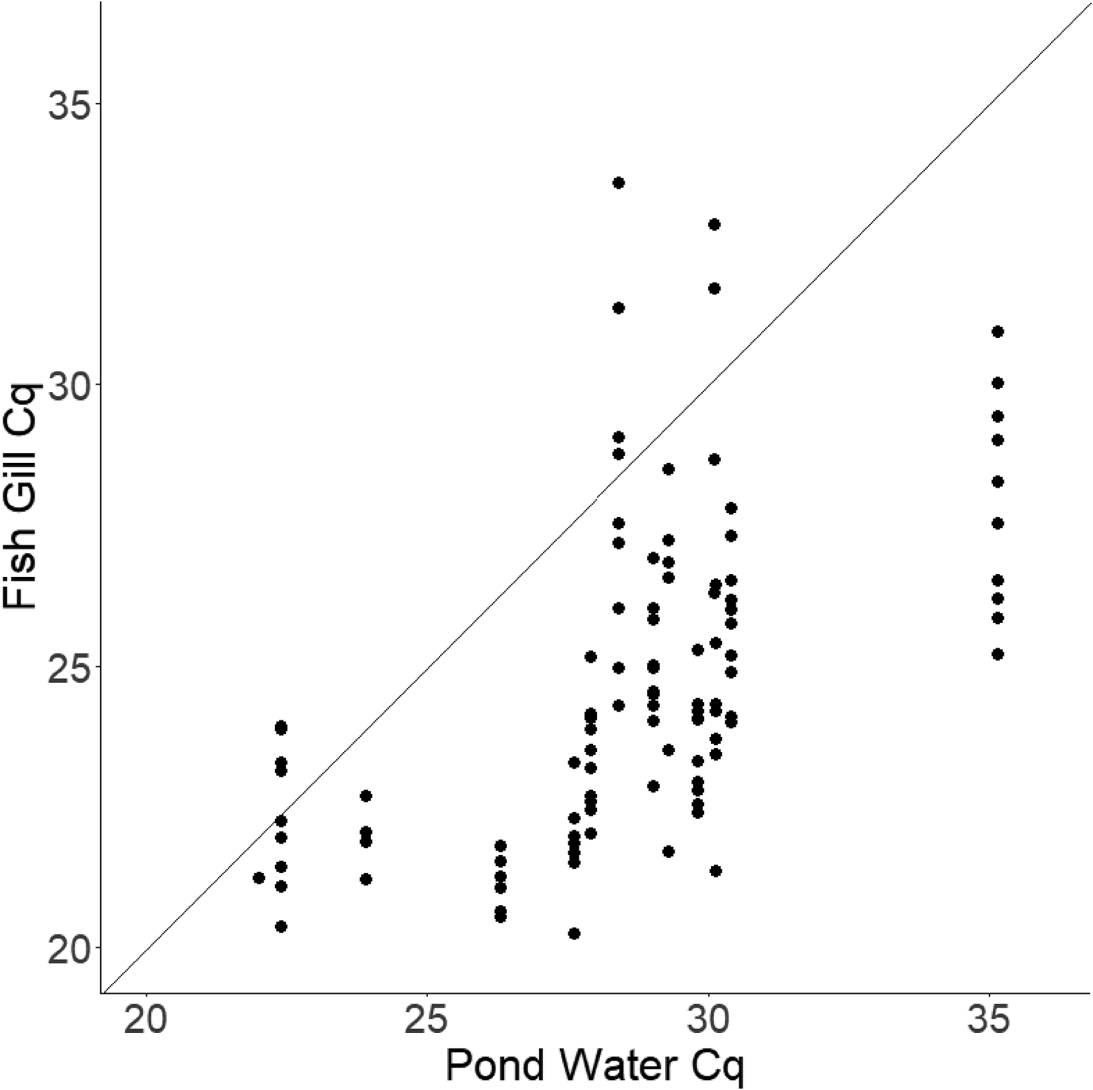
Scatterplot of pond water quantification cycle (Cq) values and Channel Catfish gill tissue Cq values. Diagonal line represents a theoretical 1:1 relationship assuming uniform and proportional infection based on parasite density in the environment.

Percent gill damage, mean gill lesions, and mean presporogonic stages showed weak correlations with estimates of pond water actinospore levels (actinospores/L; calculated from qPCR data), yielding Pearson correlations of 0.37, 0.29, and 0.10, respectively (Table 1). Correlations using pond water Cq values were slightly stronger (−0.50, −0.48, and −0.25, respectively) and were negative due to the inverse relationship between Cq values and DNA concentration in qPCR assays. Gill tissue Cq values had a similar correlation with percent gill damage (−0.50) but had slightly stronger correlations with mean gill lesions (−0.65) and mean presporogonic stages present (−0.41). Parasite DNA copy numbers (per 50 ng of genomic DNA template) in the gill tissues showed the strongest correlations with the three variables; mean gill lesions showed the strongest correlation (0.77), followed by mean presporogonic stages (0.72) and percent gill damage (0.60). Gill samples generally produced lower Cq values (i.e., higher parasite DNA loads) than water samples collected from their respective ponds (Figure 4), showing that *H. ictaluri* tended to concentrate in the gill tissues during the 7-d continual exposure.

**Table 1.** Pearson correlations for each of three Channel Catfish gill condition metrics with parasite burden metrics, presented in terms of quantitative PCR quantification cycle (Cq) and transformed to estimated actinospore equivalents per liter (pond water) or DNA copy numbers per 50 ng of template (gill tissue). Values in bold represent significant correlations at α = 0.05.

Metric	Gill damage (%)	Gill lesions	Presporogonic stages
**Pond water**			
** Cq value**	**−0.50**	**−0.48**	**−0.25**
** Actinospores per liter**	**0.37**	**0.29**	0.10
**Gill tissue**			
** Cq value**	**−0.50**	**−0.65**	**−0.41**
** DNA copies/50 ng**	**0.60**	**0.77**	**0.72**

All data analyses henceforth are based on the use of the Cq values from qPCR assays of pond water or gill tissues. In qPCR, Cq values are inversely correlated with the initial template quantity, and a 1-unit change in Cq value (1 cycle) represents an approximate doubling or halving of target DNA in the sample.

### Pond water parasite burden

**Figure 5.**
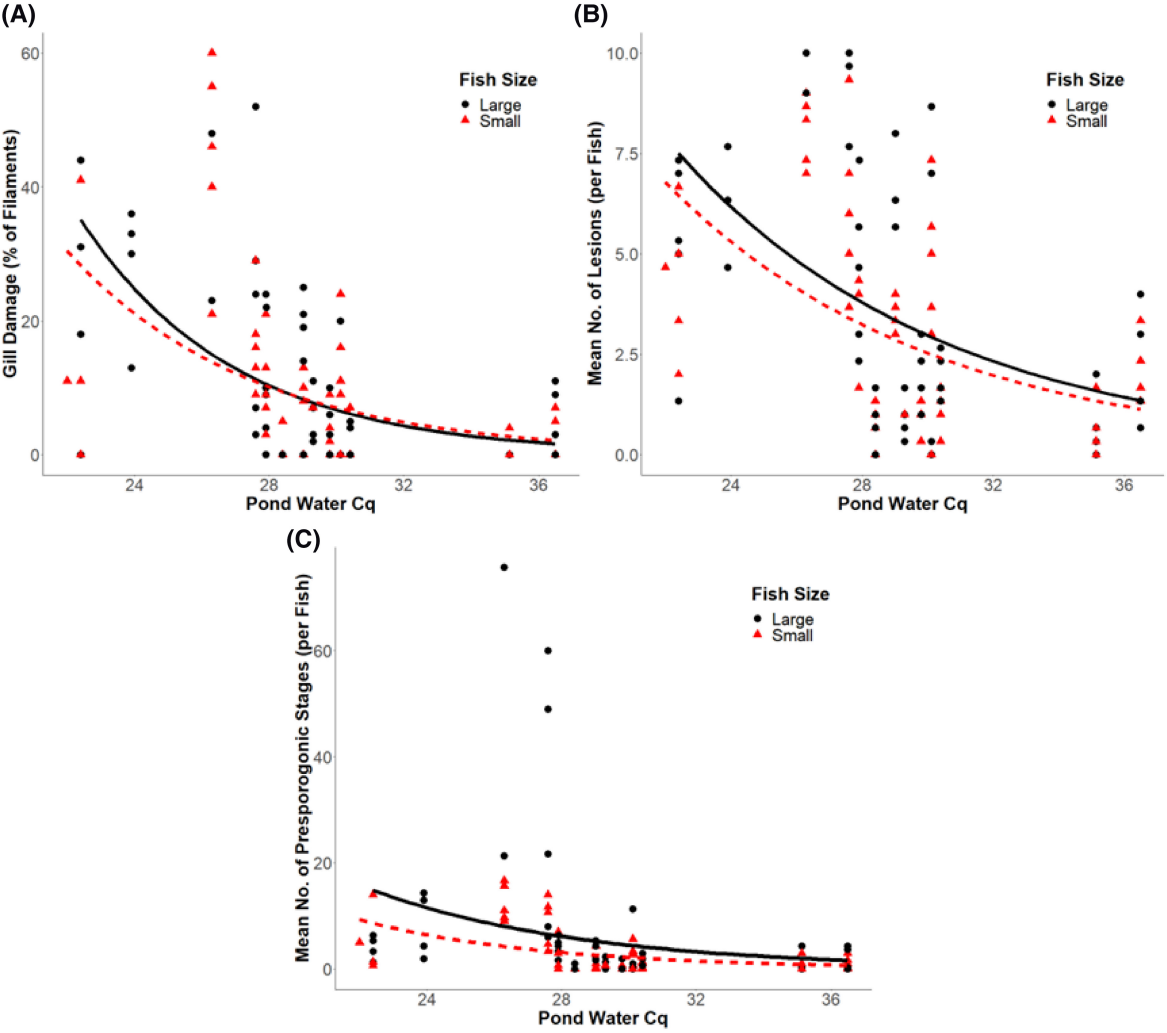
Scatterplot of (A) gill filament damage, (B) average number of lesions, and (C) average number of presporogonic stages based on *Henneguya ictaluri* actinospore concentration in the pond water (as quantitative PCR quantification cycle [Cq]) and Channel Catfish size. Lines represent estimated means from a generalized linear model with a negative binomial distribution. The Cq values have an inverse relationship with estimated DNA concentration.

The interaction between fish size and pond water Cq showed no significant influence (*P* = 0.91) on the amount of gill filament damage, yielding an odds ratio of 1.01 (95% CI = 0.95–1.07) (Figure 5A). In this original model, the parasite load in the pond water was a significant predictor across the different fish sizes (odds ratio = 0.81, 95% CI = 0.76–0.86; *P* < 0.001) while the effect of fish size was nonsignificant (*P* = 0.69) across pond water Cq values (Table 2; Figure 5A). Examination of the marginal (population-averaged) coefficients for the main effects showed a significant relationship between gill damage and pond water Cq (*P* = 0.004), where an increase of 1 Cq in pond water samples (∼50% reduction in parasite DNA) yielded a subsequent 19 ± 8.6% decrease in percent gill ­damage, on average.

**Table 2.** Exponentiated coefficient estimates from generalized linear models of parasite loads in pond water and Channel Catfish gill tissue with a negative binomial distribution, read as incidence ratios. Interaction coefficients are from global models. Marginal (population-averaged) coefficients are presented for the main-effects estimates rather than conditional coefficients. Values in parentheses represent the 95% CI for the estimate. Values in bold font are significant at α = 0.05.

	Predictor		
Variable	Cq^a^	Fish size^a^	Interaction
**Pond water**			
** Gill damage (%)**	**0.81** (**0.70–0.93)**	0.71 (0.00–200.8)	1.01 (0.95–1.07)
** Mean gill lesions (per 10 filaments)**	**1.14** (**1.02–1.28)**	1.15 (0.40–3.38)	0.98 (0.96–1.01)
** Mean presporogonic stages (per 10 filaments)**	**1.36** (**1.09–1.70)**	1.32 (0.27–6.43)	0.97 (0.92–1.03)
**Gill tissue**			
** Gill damage (%)**	**0.72** (**0.63–0.81)**	**0.04** (**0.00–0.61)**	**1.14** (**1.01–1.30)**
** Mean gill lesions (per 10 filaments)**	**0.89** (**0.84–0.93)**	2.22 (0.61–8.15)	0.96 (0.92–1.00)
** Mean presporogonic stages (per 10 filaments)**	**0.71** (**0.66–0.76)**	0.34 (0.05–2.22)	1.03 (0.95–1.12)

^a^Coefficients and CIs for main effects are marginal effects rather than conditional effects.

Observations in Figure 5A show notable deviations in gill damage between large and small fish only at the lowest Cq values. These differences at the extreme end of the data are likely due, at least in part, to the effects of survival bias. Because most fish at the highest pond water actinospore concentrations (lowest Cq values) died (Figure 1) and were not available for use in the gill tissue analyses, the surviving fish likely possessed lower parasite infestation levels than those that did not survive.

Gill lesion counts also did not appear to be significantly influenced by the interaction of fish size and pond water Cq values (*P* = 0.41). The trendline in Figure 5B shows that small fish had a lower average lesion count across all pond water Cq values; however, the effect was small, with sufficient variability to show no statistical difference between the groups (Table 2). Marginal effects for fish size were also nonsignificant (*P* = 0.79) with respect to gill lesions. Interestingly, the pond water Cq effect was significant (*P* = 0.018) but positive, suggesting an estimated increase of 14 ± 5% in mean gill lesions for each 1-unit increase in Cq value (i.e., 50% decrease in estimated actinospore equivalents).

Similar to the gill damage and lesions, fingerling size showed no significant interaction with pond water Cq values in predicting the mean number of presporogonic stages present in the gills (*P* = 0.24). Again, marginal effects of fish size were statistically nonsignificant (*P* = 0.73) while pond water Cq did show significant marginal influences (*P* = 0.006; Figure 5C). As with mean lesion counts, the marginal coefficient predicted an unintuitive 36 ± 11% increase in the number of presporogonic stages present in the gills for each 1-cycle increase in the qPCR results (Table 2). This result and the result from the lesion counts (Figure 5B) are likely due to the extreme variation in the counts at midrange Cq values.

### Gill tissue parasite burden

The interaction between Cq values from fish gill qPCR assays and fish size showed a significant influence on the percentage of damaged gill filaments (*P* = 0.031; Table 2). Coefficients of the marginal effects for the main predictor variables showed fish size (*P* = 0.021) and fish gill Cq value (*P* < 0.001) to be significant, which suggested an approximate 29 ± 6.5% decrease in gill damage for every 1-unit increase in Cq value in the assay results.

For gill damage, the interaction of fish gill Cq and fish size showed no significant influence (*P* = 0.12) with respect to the mean number of lesions present (Table 2). Marginal coefficients likewise showed a nonsignificant influence of fish size on the mean number of gill lesions per fish (*P* = 0.21). However, Cq values from the fish gill assay showed a significant marginal effect (*P* < 0.001), yielding an estimated 12 ± 2% decline in the average number of lesions present for every 1-Cq increase in the assay results.

**Figure 6.**
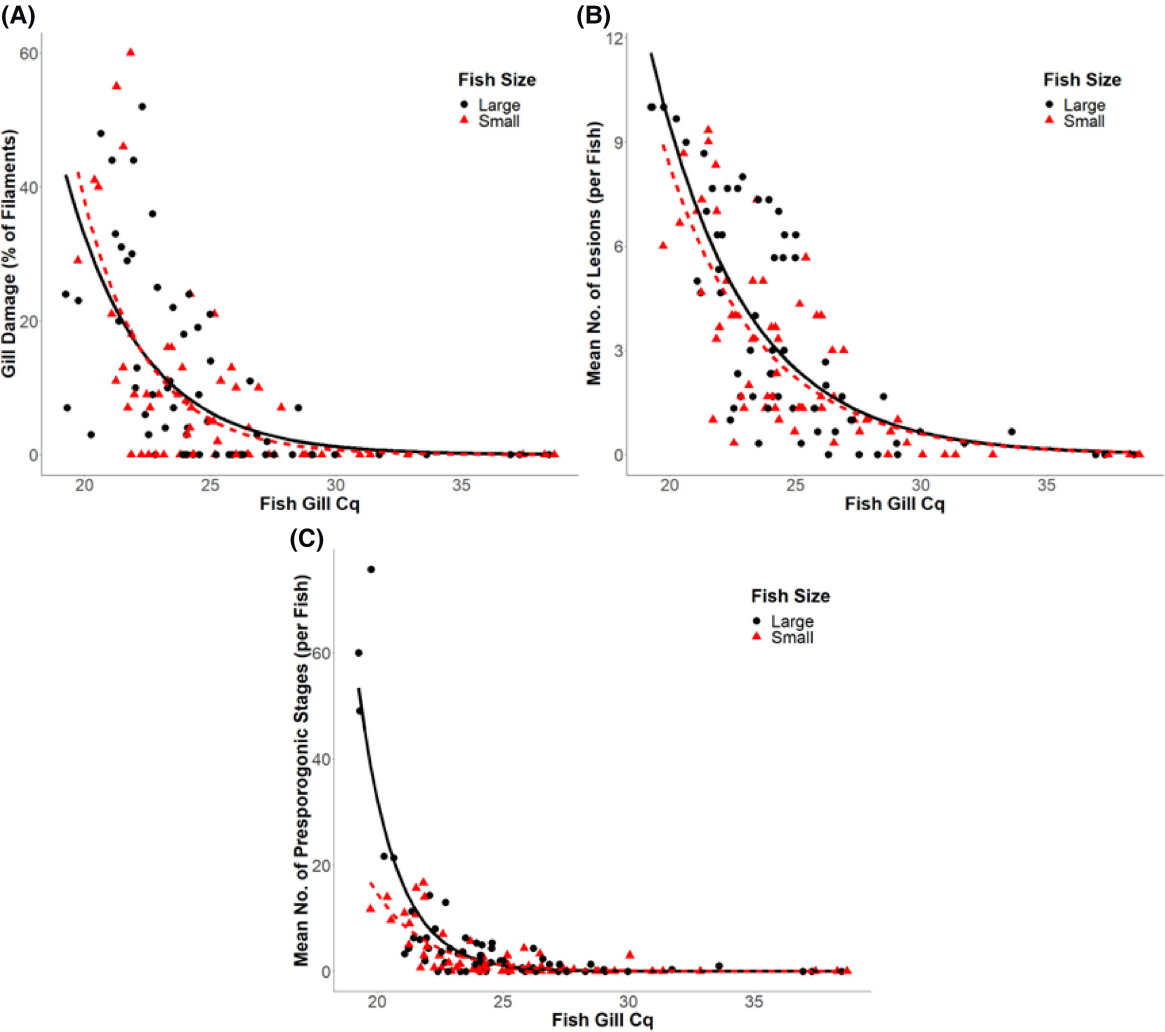
Scatterplot of (A) gill filament damage, (B) average number of lesions, and (C) average number of presporogonic stages based on *Henneguya ictaluri* actinospore concentration in the gill tissue (as quantitative PCR quantification cycle [Cq]) and Channel Catfish size. Lines represent estimated means from a generalized linear model with a negative binomial distribution. The Cq values have an inverse relationship with estimated DNA concentration.

Results from the regression of mean presporogonic stages were similar to those of previous analyses. There was no significant interaction between fish gill Cq value and fish size (*P* = 0.52; Table 2). Fish size also showed no significant marginal effects (*P* = 0.26), while fish gill Cq results were significant (*P* < 0.001). Based on the model, each increase of 1 Cq in the gill tissue assay provided a decrease of 30 ± 4% in average presporogonic stage counts (Figure 6C).

## DISCUSSION

Proliferative gill disease is one of the most important parasitic diseases affecting the U.S. catfish industry. Economic assessments of catfish farming in east Mississippi and west Alabama indicate that outbreaks of PGD result in approximately US$1.2 million in direct annual losses (Abdelrahman et al., 2023; Peterman & Posadas, 2019). These regions account for about 50% of the industry’s total production. While specific economic studies on the impact of PGD in the catfish farming regions of west Mississippi and other key areas are lacking, anecdotal reports from producers in west Mississippi suggest that PGD losses during springtime stocking exceed $2 million annually (M. J. Griffin, unpublished data). Furthermore, the economic impacts of PGD morbidity are difficult to quantify, but considering the hidden costs—including lost feed days, increased susceptibility to secondary bacterial infections, and the cost of palliative treatments—the true economic impact of PGD on catfish aquaculture in the southeastern United States likely exceeds $5 million annually. Despite the availability of several management strategies and attempts at biological and chemical control, the disease remains a major cause of industry losses, particularly in fingerling catfish (Burtle, 2000; van Hoosen, 1998; Wise et al., 2004).

The results of this study are likely influenced by survivor bias, particularly in ponds with the lowest Cq values (i.e., high actinospore concentration estimates). Postmortem autolysis in fish that died during the challenge made them unfit for histological examination, and survivorship was only assessed at the end of the 7-d exposure period. Thus, fish that survived may represent individuals that received the lowest infective exposures or they may represent individual variation in susceptibility to PGD. Results may also have been influenced by inherent variability associated with the processing and sectioning of gill tissues as well as interpretation of pathologic changes in histologic sections (Wolf et al., 2015). To mitigate variability, 10 sequential, full-length filaments were evaluated on three separate gill arches from each sampled fish. Presporogonic stage counts may have been artificially low, as some presporogonic stages were likely not observed in the single, 5-µm plane of section. For this reason, inflammatory foci were counted whether presporogonic stages were visible in the section or not.

Gill damage, number of lesions, and prevalence of presporogonic stages all showed moderate to strong correlations with the parasite burden in the gill tissue (Cq value), with Pearson correlations ranging from 0.60 (gill damage) to 0.77 (lesions). This is to be expected, as increased parasite burden in tissues should, in turn, result in more parasite-induced gill damage. However, when evaluating these relationships using pond water samples, gill damage showed only a weak correlation (0.37), while the counts for lesions and presporogonic stages show little to no relationship (0.29 and 0.10, ­respectively). This lack of relationship was possibly due to day-to-day variations in parasite concentrations over the week-long exposure that were missed by the sampling protocol or survivor bias.

The qPCR assay developed by Griffin et al. (2009) for the detection of *H. ictaluri* actinospore concentrations in pond water is the basis for a management strategy used to prevent PGD outbreaks by establishing guidelines for the “safe” stocking of fingerlings by producers. Application of the assay has set approximate thresholds for mortality risks in ponds as low (<10 spores/L), moderate (10–25 spores/L), high (25–50 spores/L), and lethal (>50 spores/L). Examination of the dose–response curves from the present study (Figure 1) provides further support for these established thresholds.

Catfish fingerlings are typically priced on a sliding scale based on size (e.g., $/inch), resulting in larger fingerlings being more expensive than smaller fingerlings and incurring a greater monetary loss with each mortality (Kumar & Engle, 2011, 2017). This means that the decision of whether to stock larger fingerlings into riskier ponds or to wait for actinospore concentrations to decline will ultimately be made on a case-by-case basis at the farmer’s discretion and should align with each farm’s management regime, production goals, and risk tolerance.

## Conclusions

Findings in this study reveal that fingerling size (18.14 kg per 1,000 vs. 54.43 kg per 1,000) has no significant impact on the outcome of PGD outbreaks and would have little utility as a management tool for controlling PGD losses in the catfish industry. While fingerling size considerations may hold relevance for other aspects of farm-specific management when aligned with production objectives, the present results do not support choosing one size-class over another as a strategy to reduce PGD-related mortality. In contrast, the results reinforce the use of qPCR to determine parasite concentrations in pond water and their validity as risk thresholds for outbreaks of PGD.

## Data Availability

Nondigital data associated with this work are available upon request. All digital data are available through the National Agricultural Library Ag Data Commons (https://doi.org/10.15482/USDA.ADC/25684692.v1).
